# A Cost-Effective Alternative for Lateral Femoral Wall Perforation in Anterior Cruciate Ligament (ACL) Reconstruction: A Case Report

**DOI:** 10.5704/MOJ.2007.023

**Published:** 2020-07

**Authors:** SY Tan, WH Leong, LH Ong, MZ Mohd-Amin

**Affiliations:** 1 Department of Orthopaedic, Universiti Malaysia Sarawak, Kuching, Malaysia; 2 Department of Orthopaedic, Sarawak General Hospital, Kuching, Malaysia

**Keywords:** ACL, endobutton, washer, blowout

## Abstract

Lateral femoral wall perforation is a rare intra-operative complication in anterior cruciate ligament (ACL) reconstruction surgery. However, it can be challenging to manage if it occurs. We share our experience on lateral femoral wall perforation managed by a large fragment washer. A 25-year-old man with right ACL injury presented with knee instability despite physiotherapy. Anterior drawer test (ADT) and Lachman test were grade 3, glide on pivot shift was positive. During ACL reconstruction, the lateral femoral wall was perforated. Due to unavailability of the rescue endobutton and budget constraint, we passed the endobutton through a washer and allowed it to sit on the washer over the lateral femoral wall. ADT and Lachman test on post-operative 6, 12 and 24 weeks were grade 1, with a negative pivot shift test. Lysholm knee score improved from 69 pre-operatively to 98 post-operatively. Conventionally, lateral femoral wall perforation can be managed by rescue endobutton, or screw and washer post technique. As this complication is rare, the rescue endobutton may not be available at all times, and the cost of the implant is also another important factor to consider. A washer can be used as an alternative technique to manage lateral femoral wall perforation in ACL reconstruction as it is not only cost-effective but also provides stable fixation with good functional outcome.

## Introduction

Lateral femoral wall perforation is a rare intra-operative complication in anterior cruciate ligament (ACL) reconstruction surgery. It is similar to over reaming of the femoral tunnel^1, 2^. Therefore, once this complication occurs, it must be managed immediately to prevent loss of graft fixation or early graft failure. Surgical management of this complication may include interference screw technique, suspensory cortical fixation with screw and washer post, suspensory fixation with the cortical button (rescue endobutton), or by hybrid fixation^3^.

We would like to share our experience in managing a lateral femoral wall perforation case by using a large fragment washer.

## Case Report

A 25 years old man presented to us with a right ACL injury. He had an alleged injury in 2015 during a football game, where his right knee was twisted with the foot planted on the field. He suffered immediate swelling over the right knee and was unable to resume the game. Upon consultation, he complained of right knee instability despite proper physiotherapy. There was no locking episode of the right knee since the injury. Clinical examination showed anterior drawer test (ADT) and Lachman test were grade 3, with a positive glide on pivot shift test. Valgus and varus stress test were negative. There was no joint line tenderness, McMurray test was negative. Magnetic resonance imaging (MRI) of the right knee showed complete right ACL tear. The patient was scheduled for right ACL reconstruction surgery.

During the ACL reconstruction surgery, the lateral femoral wall was perforated during insertion of the endobutton through the femoral tunnel due to over pulling. As there was no rescue endobutton (e.g. xtendobutton) available at that point of time in addition to budget constraint, we decided to use a large fragment washer to salvage this complication.

Thus, a small incision was made over the lateral aspect of the right distal thigh to insert the washer. We passed the endobutton through a large fragment washer (inner diameter 9mm), allowing the endobutton to hook on the washer over the lateral wall of the femur. Finally, the ACL reconstruction surgery was completed in the usual manner with a bioscrew at the tibial tunnel.

Post-operatively, he was started on physiotherapy and usual ACL rehabilitation regime. He was discharged home well on post-operative day three after wound inspection.

We followed up the patient in the clinic at post-operative two weeks, six weeks, three months, six months and one year. Clinical examination findings at pre-operative, postoperative six weeks, three months, six months and one year are documented (Table I). We assessed the functional status of the right knee by using Lysholm knee score at six months post-operatively.

**Table I:** ADT, Lachman test, Pivot shift test and Lysholm knee score for the patient at pre-operative, post-operative six weeks, three months, six months and one year

	Pre-op	Six weeks	Three months	Six months	One year
ADT	Grade 3	Grade 1	Grade 1	Grade 1	Grade 1
Lachman Test	Grade 3	Grade 1	Grade 1	Grade 1	Grade 1
Pivot Shift Test	Glide	Negative	Negative	Negative	Negative
Lysholm Knee Score	69	-	-	98	-

**Fig. 1:**
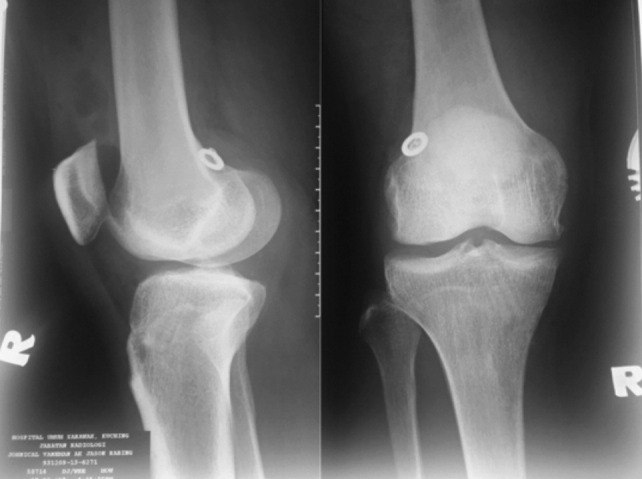
Post-operative radiographs of right knee showing placement of the endobutton and washer over the lateral femoral wall.

Lysholm knee score for the patient post-operative six months was 98 (limp component 3/5) as compared to pre-operatively, which was only 69. Anterior drawer test (ADT) and Pivot shift test both were improved from grade 3 to grade 1. Post-operative radiographs of the right knee were done to confirm the placement of the endobutton and washer on the lateral femoral wall (Fig. 1). The patient is satisfied with the outcome of the surgery.

## Discussion

**Fig. 2:**
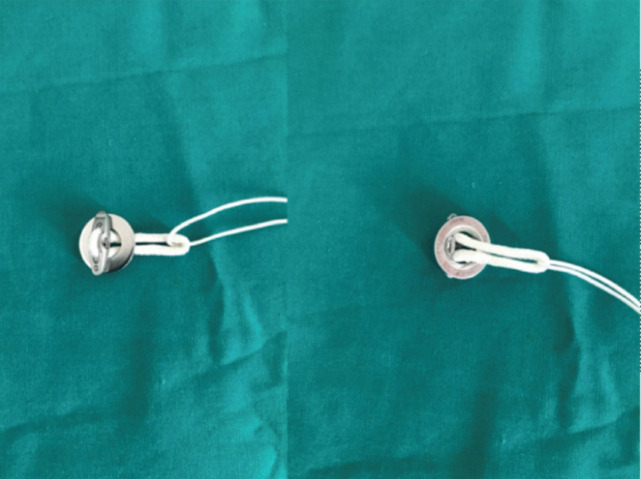
Images showing position of washer in relation with the endobutton.

**Fig. 3:**
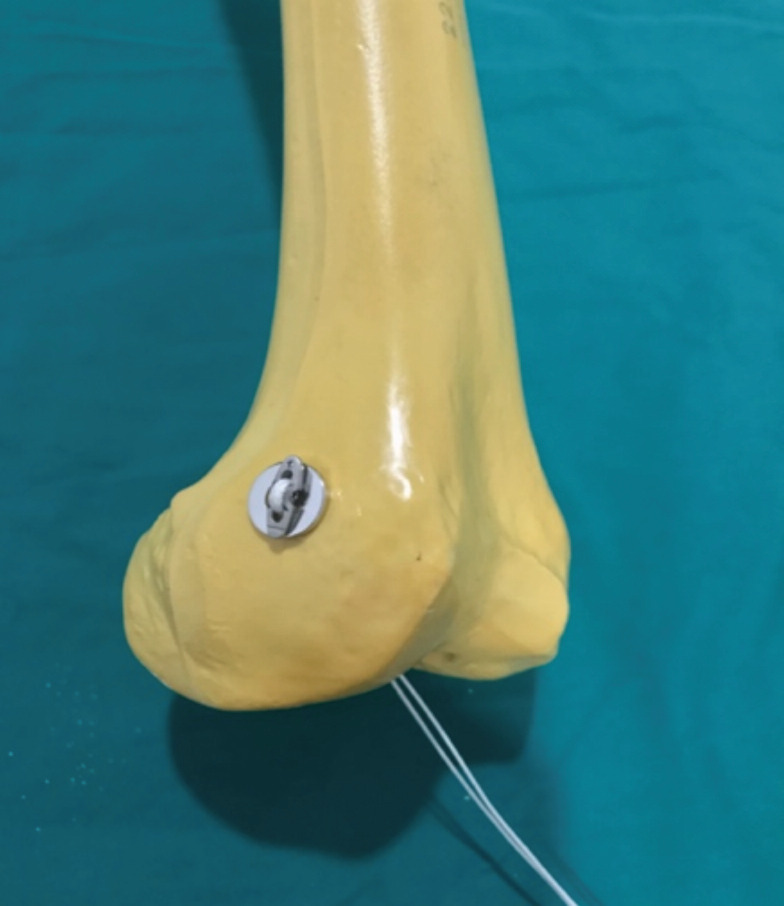
Image showing position of endobutton and washer on femur.

Lateral femoral wall perforation during ACL reconstruction surgery rarely happens. It can be avoided by careful measurements and reaming of the tunnel. Surgeons should also avoid forceful pulling during the passing of the endobutton through the femoral cortex. Surgical management of this complication includes interference screw technique, suspensory cortical fixation with screw and washer post, suspensory fixation with the cortical button (rescue endobutton), or by hybrid fixation^3^. By using the rescue endobutton, it subjects the patient to extra charges. We used a large fragment washer (inner diameter 9mm) to replace the rescue endobutton, whereby we passed the endobutton through the washer. The washer was turned around so that the flat undersurface of the washer was in contact with the flat surface of the endobutton, and the concave surface of the washer was in contact with the femoral condyle surface (Fig. 2 and 3). This helps to increase the stability of the endobutton-washer construct. With the method described, we did not sacrifice the endobutton, and the endobutton would continue to function as it was intended to.

We are sharing this experience as the functional outcome of the patient is good, as evidenced by the Lysholm knee score, which improved significantly from 69 pre-operatively to 98 post-operatively. All the components in our patient’s Lysholm knee score were improved post-operatively except for periodic limp due to a slight atrophy of quadriceps muscles. Currently, he is still on regular physiotherapy for quadriceps muscles strengthening.

Lateral femoral wall perforation usually occurs as an unforeseen event in ACL reconstruction. Therefore, rescue endobutton may not be readily available. In such event, this method can be considered as an alternative to manage the lateral femoral wall perforation in ACL reconstruction as it is not only cost-effective but also provides a stable fixation with good functional outcome.
